# Animal Models of Diabetes-Related Male Hypogonadism

**DOI:** 10.3389/fendo.2019.00628

**Published:** 2019-09-18

**Authors:** Anastasia Dimakopoulou, Channa N. Jayasena, Utsav K. Radia, Metab Algefari, Suks Minhas, Nick Oliver, Waljit S. Dhillo

**Affiliations:** ^1^Section of Investigative Medicine, Imperial College London, Hammersmith Hospital, London, United Kingdom; ^2^Department of Andrology, Hammersmith Hospital, London, United Kingdom; ^3^College of Medicine, Qassim University, Buraidah, Saudi Arabia; ^4^Department of Diabetes and Endocrinology, St. Mary's Hospital, London, United Kingdom

**Keywords:** testosterone, hypogonadism, testicular impairment, type 1 diabetes mellitus, type 2 diabetes mellitus

## Introduction

Hypogonadism is the clinical syndrome associated with low testosterone secretion in men. Hypogonadism affects ~37–57% men with diabetes mellitus (1). Male reproduction is orchestrated by the hypothalamo-pituitary-gonadal (HPG) axis, which regulates the biosynthesis of testosterone from the testes. Diabetes may cause hypogonadism through multiple mechanisms including suppression of hypothalamic gonadotrophin-releasing hormone (GnRH) secretion, or direct disruption of spermatogenesis (2). Clinical stigmata of hypogonadism include reduced libido, erectile dysfunction (ED) and reduced physical strength. This article will summarize the evidence from animal models including how diabetes affects male reproductive endocrine function and predisposes to hypogonadism.

## Epidemiology of Hypogonadism in Diabetes Mellitus

Fifty percent of men with diabetes reportedly have a reproductive disorder, including hypogonadism, defective spermatogenesis, psychosexual dysfunction due to depression associated with chronic illness, ejaculatory disorders and ED (3). The most characterized and studied manifestation of reproductive dysfunction affecting men with diabetes is erectile dysfunction. However, changes in endocrine function (4) and the central nervous system control of sexual arousal (5) also play a crucial role in the development of hypogonadism and infertility in these men.

A recent study observed that 37% (397/1089) men with type 2 diabetes mellitus (T2DM) had a serum total testosterone (TT) level <10.4 nmol/L (3 ng/ml). Among T2DM patients with low serum TT, 16.9% had primary hypogonadism (defined as LH >10 mIU/ml with TT <3.0 ng/ml). The remaining 83.1% has secondary (hypogonadotrophic) hypogonadism (defined as LH <2mIU/ml with TT <3.0 ng/ml) (1). Both primary and secondary hypogonadism are followed by rapid decline in serum testosterone in men with diabetes (6). Although, very few studies have quantified the magnitude of hypogonadism in men with type 1 diabetes (T1DM) and T2DM, a common finding between studies is the significant reduction in serum total and free testosterone in men with diabetes compared to men without diabetes (7).

The global prevalence of hypogonadism in men ranges between 10 and 40% (8), with levels declining as rapidly as 0.4–2% annually after 30 years of age (9). Diabetes mellitus affects an estimated 285 million people worldwide and this number is expected to double by 2030 (1). Consequently, hypogonadism will affect an even larger proportion of the male population in the future.

## Animal Models of Diabetes-Induced Hypogonadism

### Evidence of Hypothalamo-Pituitary Dysfunction Caused by Diabetes Mellitus

A number of studies on rodent models of T1DM have observed hypogonadism. Streptozotocin (STZ)—induced destruction of pancreatic β-cells results in the rodent model of diabetes, which is useful for the additional study of reproductive function (10). STZ-injection markedly reduces serum testosterone (T) as well as plasma luteinizing hormone (LH) (11). According to previous studies, STZ—induced diabetic rodents exhibit significant catabolic effects of insulin deficiency, undergo substantial weight loss and HH by impairing hypothalamic function (10, 11).

Insulin administration can affect hypothalamo-pituitary function. It has been observed that insulin replacement acutely increases pulsatile LH secretion in rodent diabetes models (10, 12). Steger and Kienast (11) studied comprehensively the endocrine and sexual function in adult male Sprague-Dawley rats on twice-daily insulin replacement after sham or STZ injection. STZ injection reduced plasma testosterone four-fold when compared with plasma testosterone of sham-injected rats. However, twice-daily insulin replacement fully normalized testosterone levels in male STZ-injected rats. The same authors randomized STZ-injected male rats to twice-daily insulin replacement starting either 1 day after STZ injection or 4 weeks after STZ injection. Ejaculatory function as measured by latency to ejaculation with stimulus, was fully restored to normal when insulin therapy was given without delay, but only partially restored when insulin therapy was given 4 weeks following STZ injection (11). This data suggests that insulin signaling plays a crucial role in supporting hypothalamic reproductive function. Furthermore, prolonged insulin deprivation may dampen the effectiveness of insulin treatment to restore sexual function.

Gonadotropin releasing hormone (GnRH) is secreted from the median eminence of hypothalamus to the hypophyseal portal circulation in pulses every 1–2 h, triggering corresponding pulsatile LH secretion from the pituitary gland. Since GnRH is not secreted into the peripheral circulation, pulsatile LH secretion i.e., every 5–10 min over several hours, is considered the gold-standard measure of GnRH secretion (12). When studying the effects of DM on hypothalamic GnRH function, it is important to study castrate animals to remove peripheral testosterone feedback as a confounding factor. Intriguingly, pulsatile LH secretion is significantly lower in castrate Wistar rats with STZ-induced DM when compared with castrate rats without STZ-induced DM. Pituitary sensitivity to exogenous GnRH has also been observed to be reduced by 67% (*P* = 0.001) in castrate rats with diabetes when compared with controls (12). This suggests that pituitary gonadotroph dysfunction might co-exist in diabetes-induced hypogonadism.

Altered insulin signaling in diabetes may play a detrimental role in GnRH signaling. There is evidence to suggest that central hypothalamic insulin signaling in a mouse model of diet-induced obesity (DIO) resulting in T2DM is associated with infertility (13). Knock-out of insulin receptors on GnRH-neurones led to a significant improvement in fertility rate (*P* < 0.05) and a GnRH pulsatility profile similar to that of lean mice. Control female DIO mice had an average fertility rate of 0.25 whilst the knock out of insulin receptors DIO mice had an average fertility rate of 0.67 (13). Interestingly, GnRH secretion and mean pulse amplitude in knock-out DIO mice was significantly lower compared to control mice. This was also accompanied by higher basal LH levels, determined in the morning of diestrus (13). However, this observation did not confer any improvement in fecundity. Knock-out DIO mice had a GnRH and LH profile similar to that of lean mice, suggesting that hypothalamic insulin receptor signaling may be playing an influential role in governing the reproductive axis in mice (13). No male mouse studies with DIO with or without knock out of insulin receptors have been performed so far, and remains important to determine if these would have shown similar results.

A separate study analyzed the effect of selective deletion of leptin and insulin receptors on hypothalamic pro-opiomelanocortin neurones (POMC) using a Cre-Lox recombination method to allow DNA modification. Female mice lacking leptin and insulin receptors showed reduced hepatic glucose production, decreased body weight and features suggestive of polycystic ovarian syndrome. Additionally, mice with POMC double leptin and insulin receptor knockout showed a four-fold increase in serum testosterone levels suggesting that hyperinsulinemia may be the primary factor driving increased ovarian androgen production (14). However, as this study was on female mice without diabetes, further study is required to comprehensively establish the link between insulin signaling in male with diabetes and central neuroendocrine reproductive function.

Noradrenergic signaling within the hypothalamus is implicated in the regulation of reproductive behavior (15). Hypothalamic regions associated with reproduction include the median eminence (ME), medial basal hypothalamus (MBH) and anterior hypothalamus (AH). Norepinephrine (NE) turnover is significantly reduced following STZ administration in the hypothalamic regions associated with reproductive function in adult male Sprague-Dawley rats (11). Furthermore, insulin replacement has been observed to restore NE turnover in the ME, MBH and AH following STZ treatment (11).

Hypothalamic GnRH function in T1DM may be regulated by insulin deficiency and weight loss, which is known to reduce leptin signaling required for GnRH activation (16). Studies have therefore been performed in animal models with STZ-induced diabetes (17). STZ-induced diabetic rats have significantly reduced testosterone levels when compared with controls rats subjected to caloric restriction in order to the weight loss in the STZ-injected rats (*P* < 0.05) (17). This suggests that hypogonadism associated with the STZ-induced diabetic rodent model is not merely explained by weight loss.

Targeted tissue specific insulin receptor knock-out murine models of T2DM show a spectrum of dysfunctional glucose metabolism. Liver-specific insulin receptor knock-out models are severely glucose intolerant and insulin resistant (18). Skeletal muscle specific knock-out models have increased fat mass, serum triglycerides and free fatty acids, despite having normal blood glucose and glucose tolerance (19). Adipose tissue specific knock-out models are lean, have improved glucose sensitivity and are resistant to diet-induced obesity (20). It is probable that future study of reproductive hormones in such tissue specific insulin receptor knock-out models could advance our knowledge on reproductive dysfunction in men with diabetes.

### Evidence of Testicular Dysfunction Caused by Diabetes Mellitus

In addition to impairment of hypothalamic function, diabetes may directly impair testicular endocrine function. The administration of high doses (100–200 mg/kg) of STZ to male rats induces a decrease in testosterone production in the testes (21). STZ-induced diabetes is accompanied by hypogonadism due to a reduced number of functioning Leydig cells in the testes, and impaired androgen biosynthesis within remaining functional Leydig cells (22). Insulin is expressed in the testes and regulates normal Leydig cell function by promoting DNA synthesis and steroidogenesis during puberty. In addition, insulin is crucial to Sertoli cell function, as it mediates glucose transport and lactate synthesis which is an important substrate for germ cells. Therefore, diabetes-related effects on testicular function may be a consequence of reduced insulin signaling and defective energy metabolism, although direct effects of hyperglycaemia cannot be excluded (23). In another study, the number and function of Leydig cells was markedly reduced in rats with STZ-induced diabetes when compared with controls. Reductions in testicular insulin-like growth factor I (IGF-I), androgen receptors and overall tyrosine phosphorylation were observed following STZ injection when compared with control injection, but these differences were not statistically significant (24). Further studies are needed to investigate how insulin signaling regulates testicular cell function and androgen synthesis.

Another model of T1DM is the biobreeding (BB) rat, which spontaneously develops autoimmune diabetes between 50 and 90 days (25). A study looking at gonadal dysfunction in infertile male BB Wistar rats reported a decrease in serum testosterone alongside increased Leydig cell lipid accumulation. This was also accompanied by morphological abnormalities in the Leydig cells, which had increased lipid accumulation. In addition the seminiferous tubules demonstrated increased tubular thickness, germ cell depletion and Sertoli-cell vacuolisation (26).

Recently a study investigating the effects of oral antidiabetic drugs on rodents with STZ-induced diabetes suggested a significant improvement in plasma testosterone levels following metformin treatment (27). The metformin-treated group restored testicular weight and increased serum testosterone to values similar to the control group. In contrast, pioglitazone- and sitagliptin-treated rats showed significantly lower testosterone levels compared to the control as well as the non-treated group with STZ-induced diabetes. Interestingly, the testis, epididymis and seminal vesicles of the pioglitazone- and sitagliptin-treated rats demonstrated adverse histopathological changes due to lipid peroxidation. This data suggests that metformin may be a better antidiabetic treatment option for young adults with T2DM when compared with pioglitazone- and sitagliptin. Exercise training may be another possible treatment option to restore testosterone levels, erectile function and improve insulin sensitivity in animal models with the metabolic syndrome. Rabbits fed a high fat diet exhibit glucose intolerance and decline in testosterone levels similarly to humans. After a 12 period of running on a treadmill specifically designed to accommodate rabbits, testosterone levels were found to be negatively associated with glucose levels and positively associated with the running distance (28).

### Limitations of Animal Models

The validity of the STZ rodent model of DM has recently been subject to question, as current evidence suggests that STZ can itself have cytotoxic effects on Sertoli cells, cause oxidative stress and DNA damage (29). These cytotoxic effects as well as adverse reno-hepatic effects of STZ have previously been reported (24). Furthermore, chronic experimental DM may be associated with structural damage in the hypothalamus (30). Further work, possibly using non-chemically induced diabetic rodent models such as the biobreeding (BB) rat or the non-obese mouse with diabetes may help to clarify the reproductive phenotype of diabetes.

## Summary

Diabetes causes hypogonadism through multiple mechanisms. The pathogenesis of low testosterone in animal models with diabetes includes impaired hypothalamic signaling and hypogonadotophic hypogonadism, as well as reduced testosterone production by testicular Leydig cells. Insulin replacement restores hypothalamic signaling at early stages of diabetes. Furthermore, oral antidiabetes agents such as metformin, may improve plasma testosterone levels in animal models. Further mechanistic information may help identify novel therapeutic targets for treating or even preventing diabetes-associated hypogonadism.

## Author Contributions

All authors contributed substantially to the conception and design of the work. CJ, NO, WD, MA, and SM conceptualized the study. AD, UR, CJ, and MA performed the literature search. All authors contributed to writing the manuscript. All authors are in agreement to be accountable for all aspects of the work in ensuring that questions related to the accuracy or integrity of any part of the work are appropriately investigated and resolved.

### Conflict of Interest Statement

CJ is Chief Investigator on the NIHR Health Technology Assessment-funded Testosterone Efficacy & Safety (TestES) Consortium. CJ received an honorarium for debating the safety of testosterone therapy at a meeting organized by Society of Endocrinology sponsored by Besins healthcare. The remaining authors declare that the research was conducted in the absence of any commercial or financial relationships that could be construed as a potential conflict of interest. The handling editor declared a past co-authorship with one of the authors CJ.
